# Duodenal Perforation After Blunt Trauma: CT and Operative Correlation of an Unexpected Finding

**DOI:** 10.7759/cureus.115166

**Published:** 2026-08-25

**Authors:** Fatim Zahra Hanine, Kenza Fathellah, Kamal Benzidane, Omar Kacimi, Khalid Elhattabi

**Affiliations:** 1 Radiology, Ibn Rochd University Hospital, Casablanca, MAR; 2 General Surgery, Ibn Rochd University Hospital, Casablanca, MAR

**Keywords:** blunt trauma, computed tomography, duodenum, intestinal perforation, laparotomy

## Abstract

Duodenal injury from blunt abdominal trauma is uncommon and often difficult to diagnose early due to its retroperitoneal location and nonspecific clinical presentation, which delays proper management and increases morbidity and mortality. A 32-year-old man sustained blunt abdominal trauma in a motor vehicle accident. CT showed active contrast extravasation from the first duodenum with moderate hemoperitoneum and associated American Association for the Surgery of Trauma (AAST) III liver lacerations. Exploratory laparotomy confirmed duodenal perforation treated by primary repair and feeding jejunostomy. Hepatic injuries were managed conservatively. Postoperative course was uneventful. Oral feeding was resumed three weeks later after removal of the jejunostomy tube. This case highlights the diagnostic challenges of blunt duodenal injuries and emphasizes the pivotal role of contrast-enhanced CT in their early detection. Direct imaging signs, although infrequent, are highly specific and should prompt urgent surgical management. A review of the literature confirms that early diagnosis, accurate injury grading, and tailored surgical repair are critical determinants of outcome, while delays beyond 24 hours markedly increase complication rates and mortality. High clinical suspicion, appropriate imaging, and timely operative intervention are keys to improving outcomes in blunt duodenal perforation.

## Introduction

Trauma is an evidently frequent cause of morbidity and mortality worldwide. Our Emergency Radiology Department, in the trauma center of a University Hospital, is no exception to the major hubs of trauma, accidents, and disasters. Abdominal trauma, and the blunt kind, is one of the most frequently admitted cases in our facility and many regions. However, duodenal injury in this context is very rare, accounting for approximately 3.7-5% of abdominal trauma [1]. Its diagnosis is frequently delayed due to subtle clinical presentations and non-specific radiological findings [2]. This results in higher chances of complications, prolonged hospital stays, and mortality, highlighting the importance of early detection and management [3].

Established CT findings of duodenal injury include bowel wall thickening, mural discontinuity, extraluminal air, retroperitoneal fluid, mesenteric fat stranding, and contrast extravasation into the retroperitoneum or peritoneal cavity. However, intraluminal intravenous contrast extravasation has rarely been described in association with traumatic duodenal perforation [2]. This case highlights an unusual CT finding that may represent an associated imaging marker of severe full-thickness duodenal injury.

## Case presentation

The patient was a 32-year-old male patient with no particular past medical or surgical history and no medication use or anticoagulation therapy. He was admitted to the emergency department for blunt abdominal trauma due to a motor vehicle injury three hours prior.

He presented with diffuse abdominal pain, predominantly in the epigastric region without associated vomiting or externalized bleeding. The physical exam found a conscious and oriented patient. Hemodynamic assessment showed tachycardia (110 beats per minute; reference range: 60-100 beats per minute), normal blood pressure (105/70 mmHg), and normal oxygen saturation on room air. Abdominal examination found generalized tenderness with guarding predominantly in the upper region, without abdominal distension or visible signs of trauma such as ecchymosis or seatbelt marks.

Initial management and workup included the first steps of conditioning (monitoring of vital signs, peripheral venous line access, and laboratory investigations: complete blood count, coagulation profile, serum electrolytes, blood typing, and arterial blood gas). The results are shown in Table 1.

**Table 1:** Initial laboratory findings and arterial blood gas analysis Initial laboratory findings revealed leukocytosis, and elevated inflammatory markers. Arterial blood gas analysis showed no significant acid–base disturbance.

Parameter	Value	Reference range
Hemoglobin (g/dL)	13.5	13–17
White blood cells (/mm³)	14,200	4,000–10,000
Platelets (/mm³)	241,000	150,000–400,000
C-reactive protein (CRP; mg/L)	18	<5
Sodium (Na⁺; mmol/L)	138	135–145
Potassium (K⁺; mmol/L)	3.9	3.5–5.0
Creatinine (mg/dL)	0.9	0.6–1.3
Urea (g/L)	0.35	0.15–0.45
Prothrombin time (PT)	90%	70-100%
pH	7.37	7.35-7.45
PaCO2 (mmHg)	36	35-45
PaO2 (mmHg)	95	80-100
HCO3^-^ (mmol/L)	21	22-26
Base excess (mmol/L)	-3	-2 to +2

A Focused Assessment with Sonography for Trauma (FAST) ultrasound revealed peritoneal free fluid, and eventually a whole-body CT.

**Figure 1:**
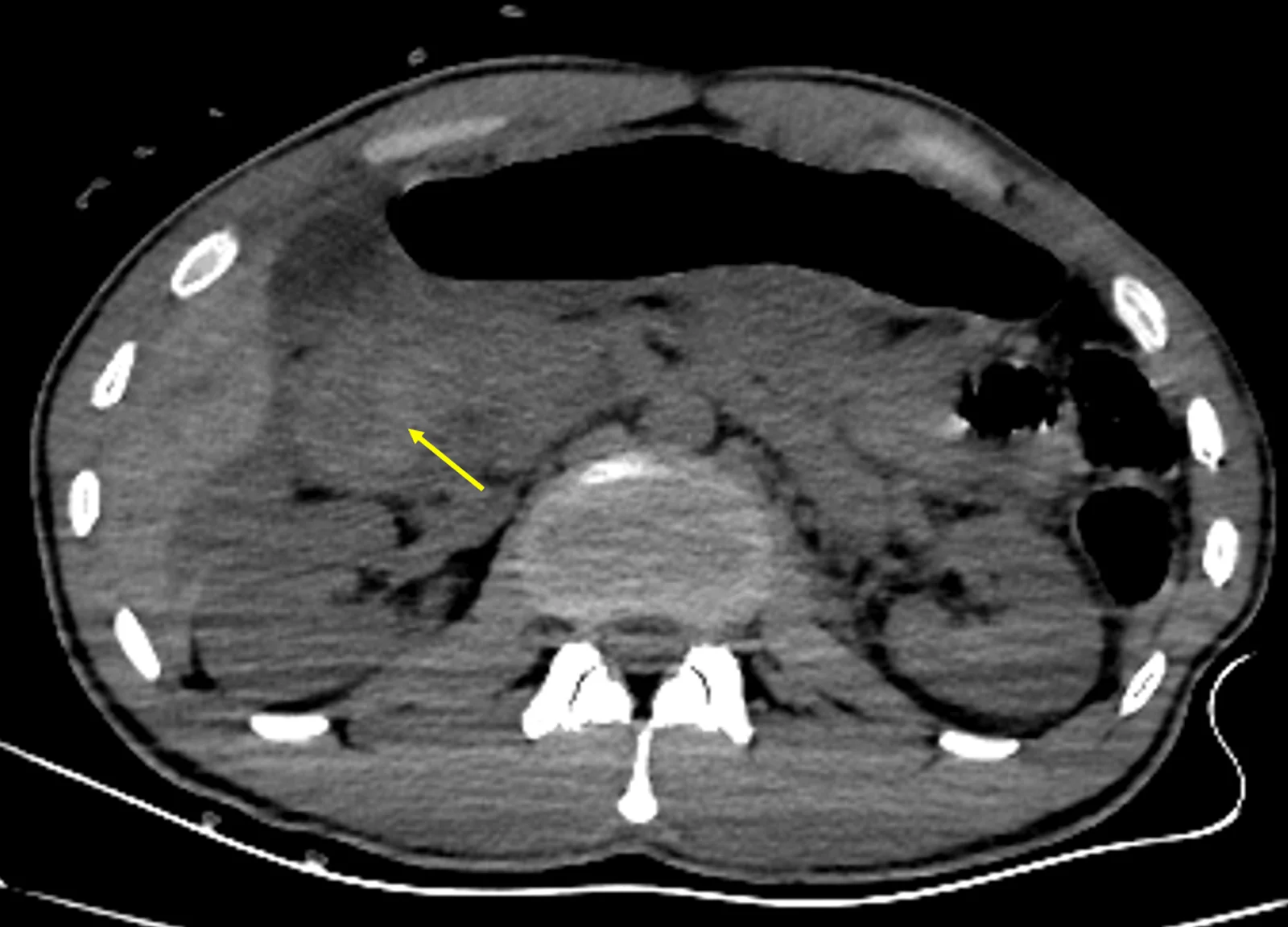
Imaging findings from abdominal non-contrast CT Axial abdominal CT without contrast shows focal spontaneous hyperdensity of the duodenal wall at D1 (yellow arrow).

**Figure 2:**
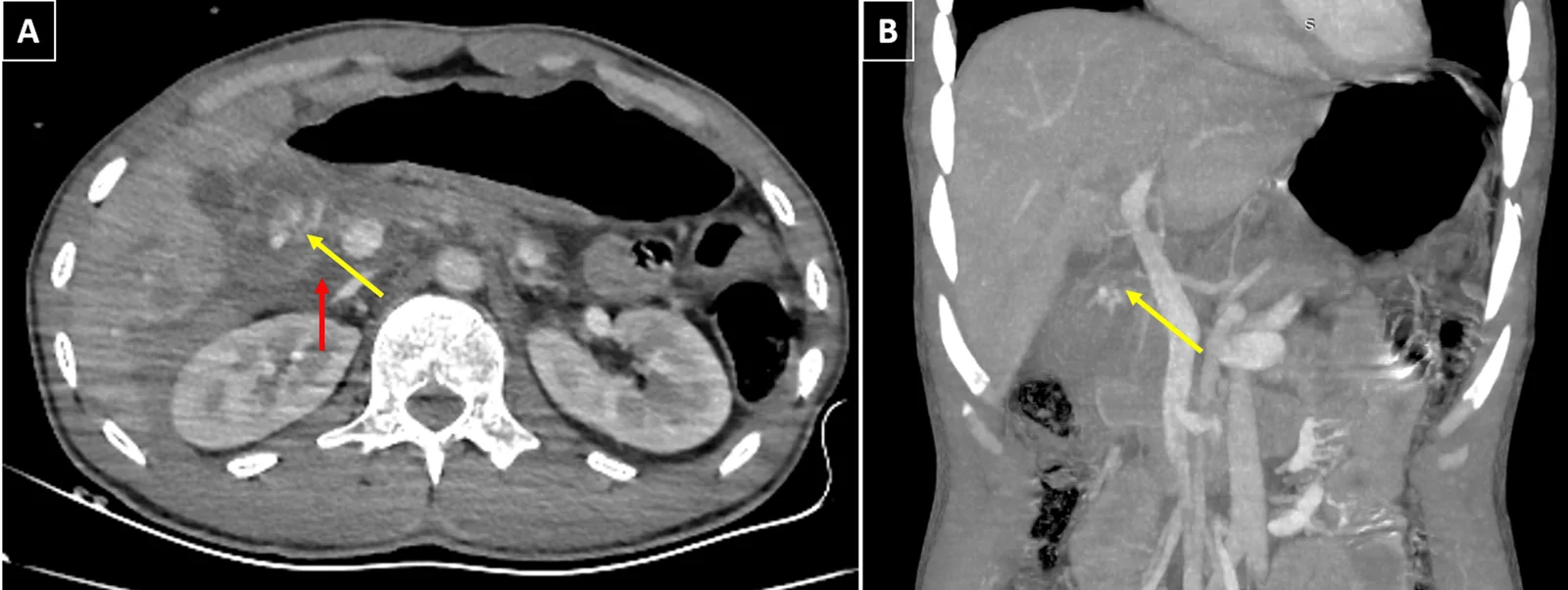
Imaging findings from abdominal post-contrast CT (arterial phase) A: Axial abdominal CT post IV contrast at arterial phase shows contrast endoluminal extravasation (yellow arrow) and adjacent free fluid (red arrow); B: Maximum Intensity Projection (MIP) of coronal reconstruction shows contrast endoluminal extravasation (yellow arrow).

**Figure 3:**
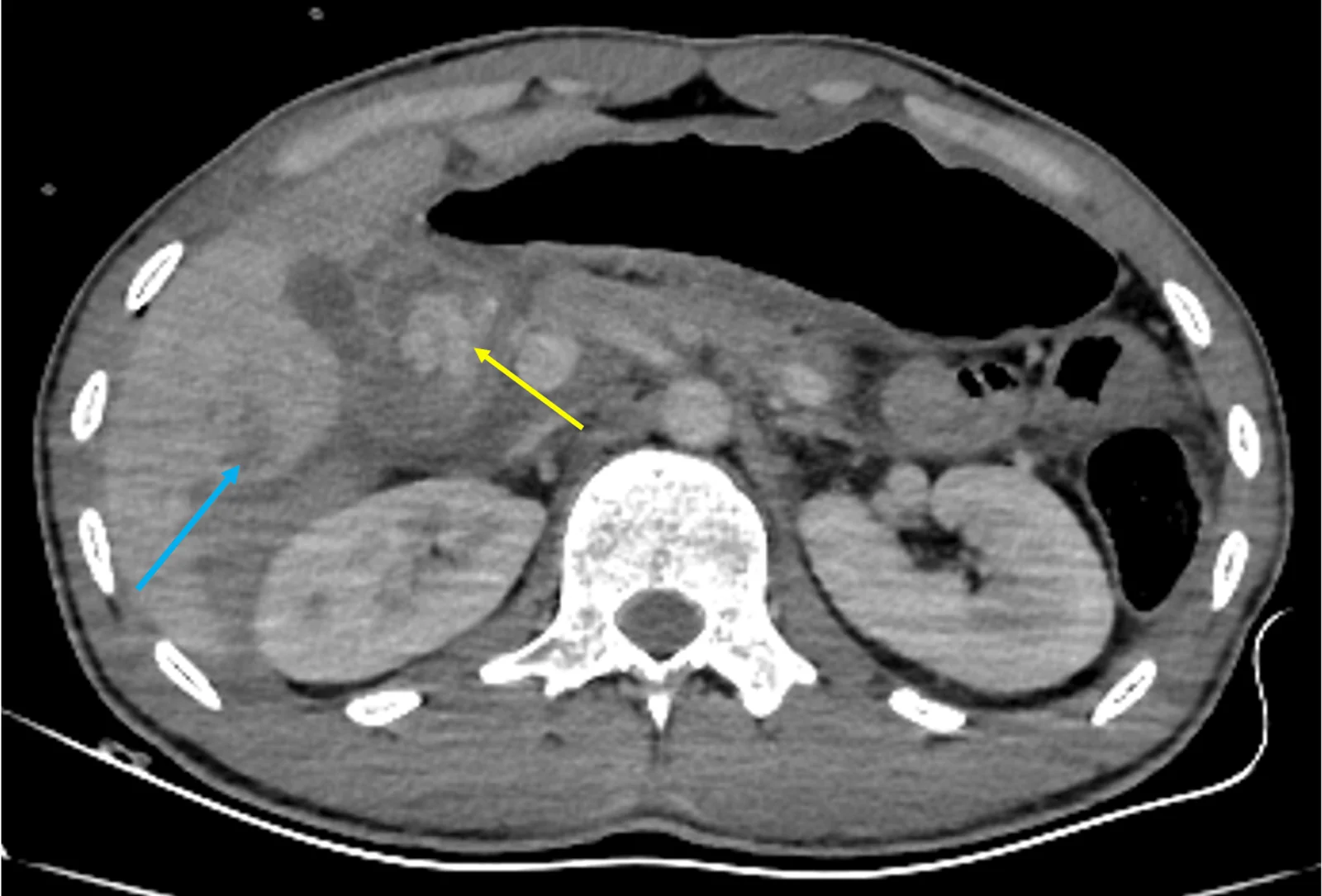
Imaging findings from abdominal post-contrast CT (portal phase) Axial abdominal CT post IV contrast at portal phase shows contrast endoluminal extravasation (yellow arrow), and segment VI hepatic laceration.

The imaging findings on the CT scan noted focal spontaneous hyperdensity of the duodenal wall at D1, endoluminal intravenous contrast extravasation within the first duodenum at arterial and portal acquisition, adjacent free fluid, moderate haemoperitoneum. We also reported hepatic lacerations grade American Association for the Surgery of Trauma (AAST) III and iliac fractures, but no pneumoperitoneum, no clear duodenal wall disruption and no retroperitoneal fluid or collection (Figures 1-3).

Oral contrast was not administered considering the state of the patient.

**Figure 4:**
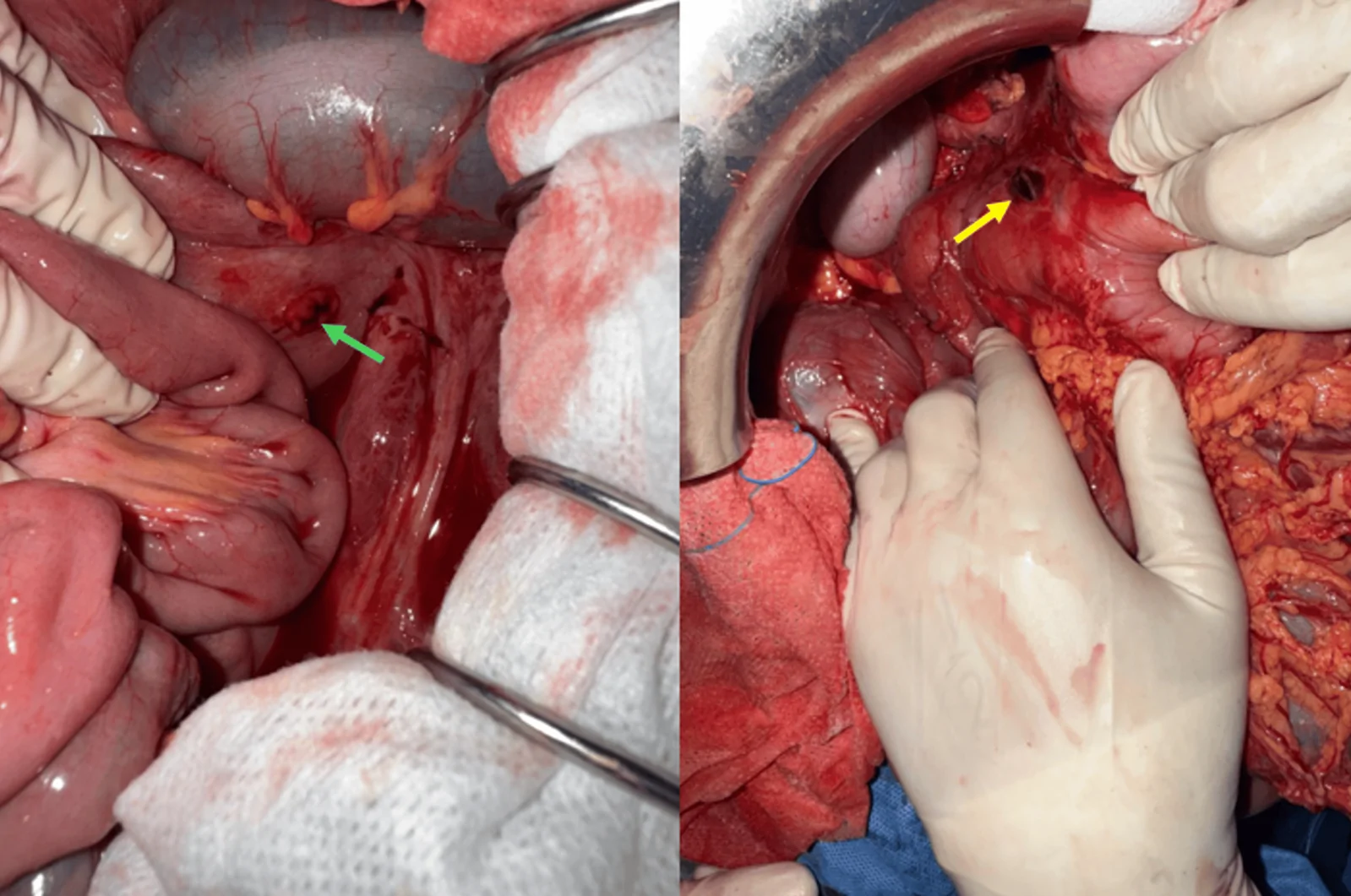
Operative findings Preoperative image showing perforation of D4 (green arrow) and D1 (yellow arrow).

The patient underwent brief stabilization in the resuscitation unit before being transferred to the operating room. Abdominal access was achieved through a midline supra- and infra-umbilical incision. Upon entering the peritoneal cavity, a moderate hemoperitoneum was identified and evacuated. A segment V hepatic laceration was identified intraoperatively, with no active bleeding requiring direct intervention, and was managed conservatively and no specific hemostatic procedure was documented. Exploration of the gastrointestinal tract revealed two perforations: the first on the anterior surface of the duodenal bulb (D1), and the second on the posterior wall of the fourth portion of the duodenum (D4). The D1 perforation was intraperitoneal, consistent with its anterior location, while the D4 perforation, although on the posterior wall, did not produce significant retroperitoneal fluid due to its small size and the sealing effect of surrounding retroperitoneal structures. The remainder of the digestive tract showed no abnormalities (Figure 4).

The injury was initially classified as AAST grade III. However, precise retrospective confirmation was not possible because the operative report did not document the defect size or percentage of circumferential involvement. Consequently, a precise lesion-by-lesion application of the AAST criteria could not be performed, representing a limitation of this case report.

A Kocher maneuver was performed to mobilize the duodenum and allow systematic inspection of D2 and D3, while the distal duodenum was explored through mobilization of the ligament of Treitz.

Retrospective review of the CT examination did not reveal a definite abnormality at the D4 site, indicating that this injury was radiologically occult.

The procedure consisted of primary repair of both perforations using interrupted 2-0 Vicryl sutures, followed by the creation of a feeding jejunostomy to allow early enteral nutrition and promote optimal healing of the repairs. A subhepatic drain and a left subphrenic drain were placed using a Salem tube.

A feeding jejunostomy was created despite primary repair because the patient had two anatomically distinct duodenal perforations associated with high-energy blunt abdominal trauma and concomitant hepatic injuries. Given the multiplicity of injuries and the anticipated delay in oral feeding, secure enteral nutritional support was considered appropriate while protecting the duodenal repairs.

The postoperative course was uneventful. No complications were noted and no contrast imaging was needed. Enteral feeding through the jejunostomy tube was initiated on postoperative day two after the return of bowel function.

The patient was discharged on postoperative day four in clinically stable condition. He didn't present with any fever, abdominal pain, or signs of peritonitis. We also noted the patient's satisfactory tolerance of jejunostomy feeding. Home enteral nutrition was continued according to the prescribed regimen. Follow-up was arranged to monitor for delayed anastomotic leakage, intra-abdominal infection, tube-related complications, and nutritional intolerance. At the three-week follow-up visit, the jejunostomy tube was removed and oral feeding was resumed after clinical recovery and satisfactory tolerance was established.

## Discussion

Epidemiology and lesional mechanism

Epidemiology

Duodenal injuries (DI) are a fairly rare occurrence, representing only 2-5% of trauma patients. Similarly to other abdominal injuries, they can be classified as blunt or penetrating, with penetrating mechanisms accounting for the majority of cases. Most DI result from penetrating trauma and only a small portion are due to blunt trauma. The latter are predominantly caused by motor vehicle collisions (85%), with falls and assaults being less common (Table 2) [1,2].

**Table 2:** Mode of injury [3,4]

Mode of injury	Flint et al.	Bozkurt et al.
Blunt: (Motor vehicle/Direct blow or crush/Miscellaneous)	23%	38%
Penetrating: (Gunshot wound/Stab/Miscellaneous)	77%	62%

Lesional Mechanism

When it comes to blunt trauma, DIs can result from either a (1) crushing force: causing direct compression of the bowel wall; (2) shearing force: tearing a fixed portion by acceleration-deceleration mechanisms, or (3) bursting force: rupturing the bowel wall due to an acute increase in the intraluminal pressure.

In consequence, we can note four major types of injury; hematomas and contusions, perforations (involving <20% of the luminal circumference), laceration (20-70% of the luminal circumference), and disruption (involving >70% of the luminal circumference) [1].

Clinical signs

As we focus on the duodenal perforation due to blunt trauma, the clinical presentation of DI altogether remains subtle and nonspecific. This is largely due to its retroperitoneal location, which may delay diagnosis.

Some of the most described signs to look for are abdominal pain; particularly epigastric, and possible nausea, vomiting, or hematemesis.

External markers of trauma such as seat-belt signs, ecchymosis, or penetrating wounds should heighten suspicion.

Physical findings range from mild abdominal tenderness to signs of peritonitis, which are more suggestive of duodenal perforation [5,6].

Imaging findings

Because physical examination may be initially unremarkable, a high index of suspicion is required, especially following high-energy blunt trauma or penetrating injuries.

Hemodynamic instability, peritonitis, or evisceration mandates immediate exploratory laparotomy, preceded by a FAST to identify free abdominal fluid [1].

In stable patients, contrast-enhanced multidetector CT is the diagnostic modality of choice for the evaluation of suspected DI, as it may reveal retroperitoneal air or fluid collections. These findings are highly suggestive of perforation but only clear duodenal wall disruption remains specific [7].

Although oral contrast may increase detection of luminal extravasation, it is not routinely administered in trauma protocols [8].

CT demonstrates high diagnostic accuracy for bowel and mesenteric injuries; however, DIs, particularly following blunt trauma, are frequently subtle and may be initially overlooked. Reported miss rates of up to 27% have been attributed to the retroperitoneal location of the duodenum and the nonspecific nature of early imaging findings [9].

Upper gastrointestinal contrast studies may be useful in hemodynamically stable patients with delayed or equivocal symptoms but should not replace CT as the initial diagnostic modality. Diagnostic peritoneal lavage has limited sensitivity for retroperitoneal DIs and is now rarely employed. Despite advances in imaging, surgical exploration remains the definitive diagnostic standard, particularly when CT findings are equivocal and clinical suspicion for duodenal perforation persists (Table 3) [2,10].

**Table 3:** Multidetector CT findings in duodenal traumatic perforation [11,12]

Direct signs	Indirect signs
Focal wall discontinuity	Duodenal wall thickening
Extraluminal air (often retroperitoneal)	Abnormal luminal configuration
Contrast extravasation when oral contrast is used	Adjacent fat stranding
Localized peri duodenal fluid collections	Unexplained retroperitoneal fluid
	Associated pancreatic or vascular injuries

Potential Imaging Correlate of Duodenal Perforation

This brings us to our case, where we have only found peri-duodenal fluid as a direct sign. However, this sign can be explained by the adjacent hepatic lacerations. The absence of retroperitoneal fluid can be explained by the location of the perforations being in the intraperitoneal portion of the duodenum (D1). The D4 perforation did not produce significant retroperitoneal fluid due to its small size and the sealing effect of surrounding retroperitoneal structures.

To date, intravenous contrast endoluminal extravasation has rarely been described in the literature as a radiological sign of duodenal perforation. In the present case, laparotomy subsequently confirmed two full-thickness defects close to where focal accumulation of IV contrast within the duodenal lumen was described.

**Figure 5:**
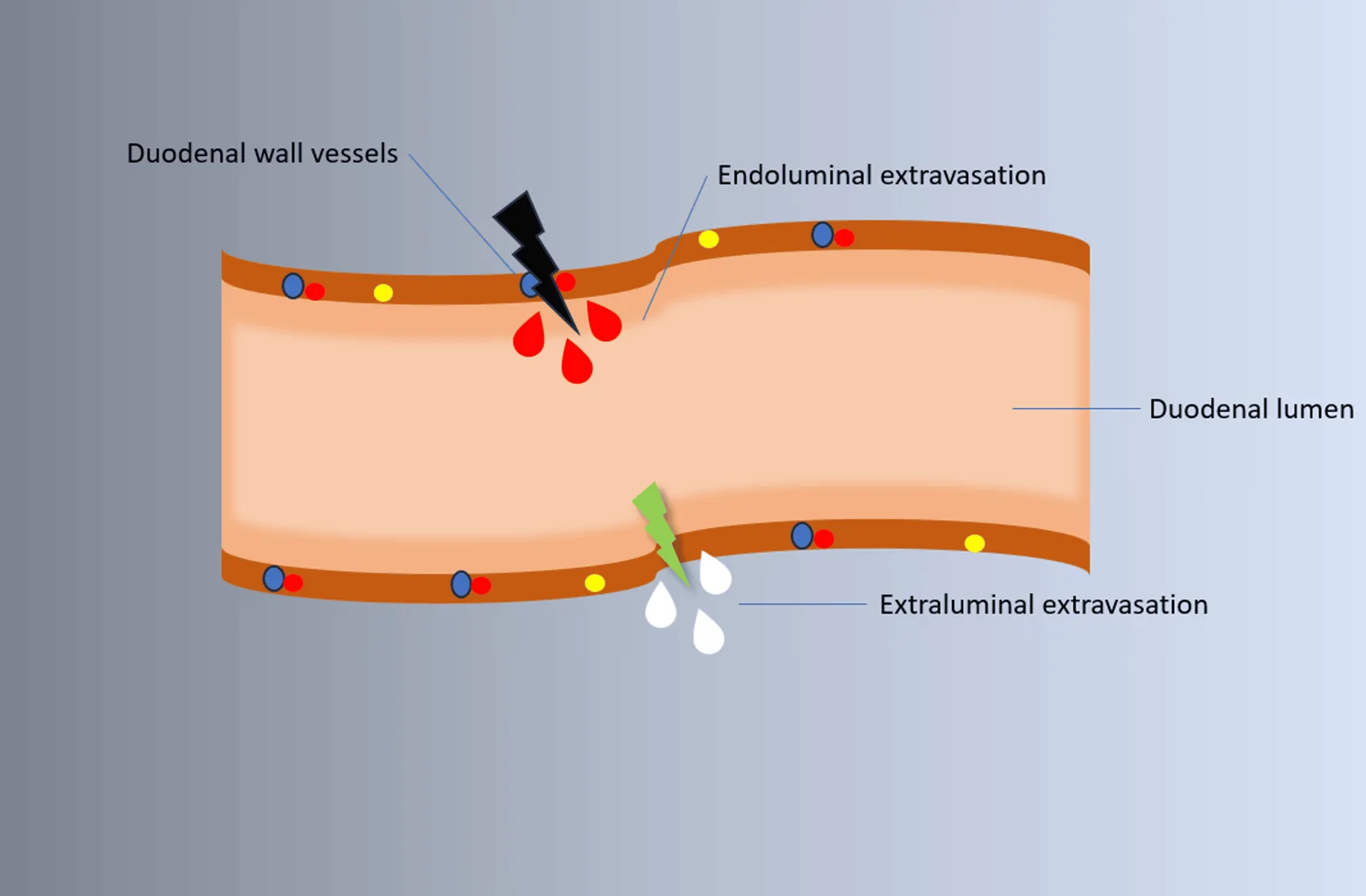
Illustration of IV contrast leak mechanisms We distinguish the mechanisms of intra-veinous contrast extravasation inside (black flash) and outside (green flash) the lumen of the bowel. Image credit: Created using Microsoft Powerpoint (Microsoft Corp., Redmond, WA, USA).

This observation may be explained by transmural disruption of the duodenal wall and intramural vessels, allowing vascular contrast to leak directly into the bowel lumen. Although this mechanism differs from the classic concept of extraluminal enteric contrast leakage, it represents a plausible physiopathological phenomenon (Figure 5).

This finding can also reflect active hemorrhage from disrupted intramural or mucosal vessels into the duodenal lumen. In the present case, the extravasation was anatomically concordant with the surgically confirmed D1 perforation.

While this finding remains observational and cannot be generalized, unexplained intraluminal contrast on portal venous phase CT in the appropriate traumatic context may represent an additional indicator of duodenal perforation and should be interpreted with caution.

Classification

DIs are classified from grade I to V according to the AAST Organ Injury Scale, which stratifies injuries based on anatomic extent and severity.

Although AAST grade alone does not predict mortality, we must mention that accurate injury grading is essential for guiding operative versus non-operative strategies [13].

The World Society of Emergency Surgery (WSES) classification on the other hand, divides duodenum injuries into four classes and three grades, taking both AAST classification and haemodynamic status into consideration (Table 4) [14].

**Table 4:** Combined WSES and AAST duodenal injury scale WSES: World Society of Emergency Surgery; AAST: American Association for the Surgery of Trauma [14].

Grade	WSES class	AAST	Description of injury
Minor	WSES class I	I	- Hematoma involving a single portion of duodenum - Laceration: partial thickness, no perforation
Moderate	WSES class II	II	- Hematoma involving more than one portion - Laceration with disruption of less than 50% of circumference
Severe	WSES class III	III	- Disruption 50–75% of circumference of D2 - Disruption 50–100% of circumference of D1, D3, or D4
		IV	Disruption >75% of circumference of D2 Involving ampulla or distal common bile duct
		V	Massive disruption of duodenopancreatic complex
			Devascularization of duodenum
	WSES class IV	Any	Any degree of lesion with hemodynamic instability

Treatment and management

**Figure 6:**
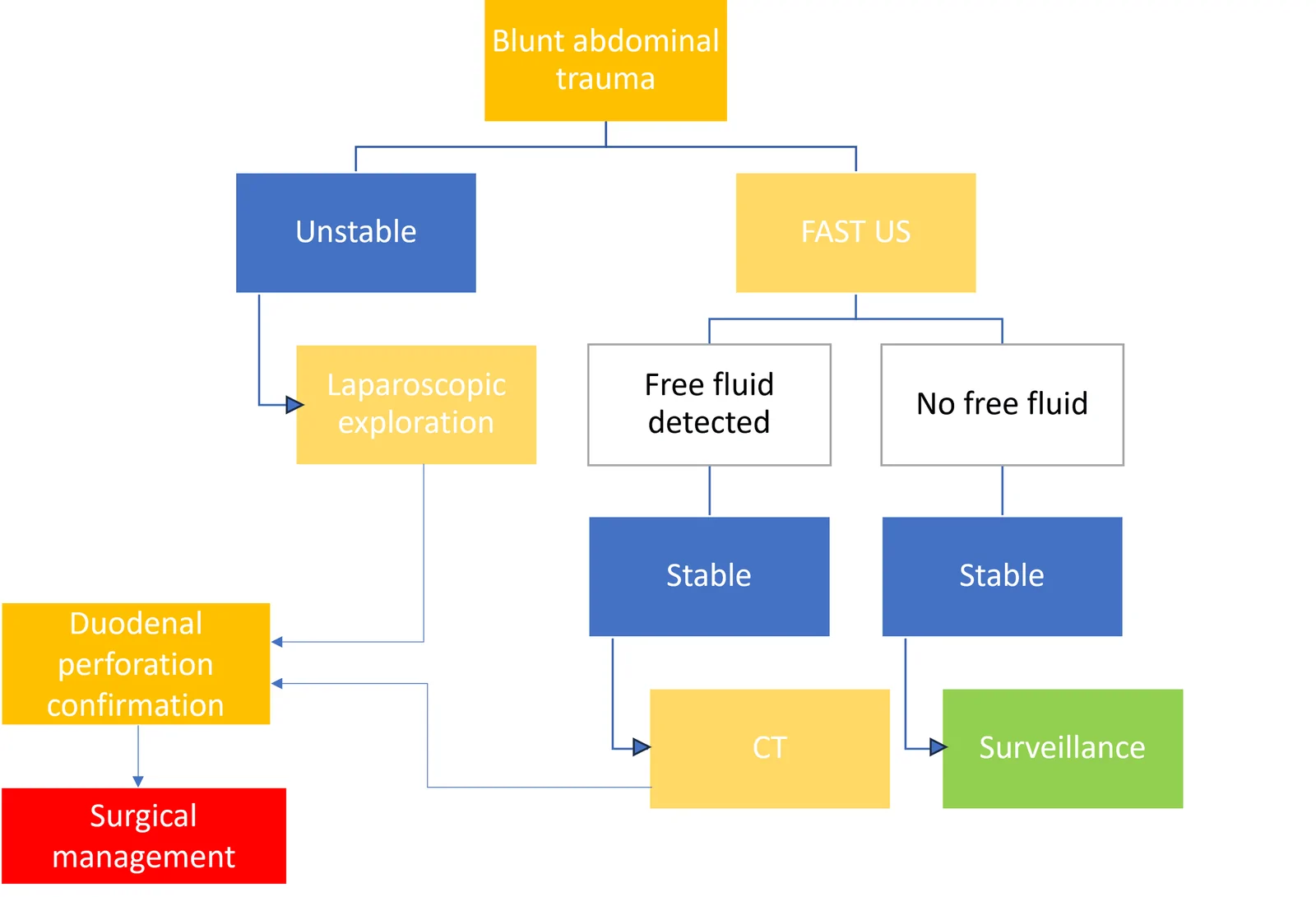
Management of traumatic duodenal perforations Image credit: Created by the authors using Microsoft PowerPoint (Microsoft Corp., Redmond, WA, USA).

Traumatic duodenal perforations require tailored surgical management based on injury severity, hemodynamic status, and associated lesions (Table 5 and Figure 6) [2,10,15,16]

**Table 5:** Management and repair options for duodenal injury [2,10,15,16]

Management strategy	Indication/Role
Non-operative management	Isolated duodenal hematoma without perforation
Primary duodenal repair (simple suturing)	Most duodenal perforations and lacerations
Serosal or partial-thickness repair (interrupted sutures)	Minor injuries without full-thickness disruption
Nasogastric decompression	Protection of duodenal repair
Extraluminal drainage	Selective adjunct for complex injuries
Pyloric exclusion with gastrojejunostomy	High-grade injuries or associated pancreatic trauma
Duodenal diversion techniques	Selected complex or high-risk repairs
Feeding jejunostomy	Postoperative nutritional support
Roux-en-Y duodenojejunostomy	Significant tissue loss or ampullary involvement
Segmental duodenal resection with reconstruction	Non-repairable localized injuries
Damage control surgery	Hemodynamically unstable patients
Staged pancreaticoduodenectomy (Whipple procedure)	Massive duodenopancreatic disruption (delayed setting)
Endoscopic closure techniques (clips or sutures)	Highly selected, contained perforations

Outcomes and prognosis

Prognosis

Duodenal trauma carries substantial morbidity (22-27%) and mortality (5-30%), which increase with injury severity and delayed diagnosis. Mortality rises from approximately 8% in grade I injuries to nearly 60% in grade V injuries [17].

Early deaths are primarily due to hemorrhage, whereas delayed mortality is related to fistula formation, sepsis, and multiorgan failure. A diagnostic delay exceeding 24 hours is a major predictor of poor outcome, with mortality increasing nearly fourfold. [18]

Complications

Post-operative complications are not uncommon. They can vary from missed injuries, intraabdominal abscess, duodenal fistula, duodenal obstruction, recurrent pancreatitis or bleeding [1].

Limitations

We recognize that this report has several limitations. We cite the single-patient nature of the study and observational nature of this radiological finding with its surgical correlate. We can consider the plausibility of a new indicator for duodenal perforation being endoluminal intra-veinous contrast extravasation, however, it cannot be generalized. Further studies with larger cohorts are necessary to validate its diagnostic value and reproducibility in the setting of duodenal trauma.

## Conclusions

Duodenal perforation following blunt abdominal trauma remains a rare but potentially life-threatening injury, often missed in early evaluation due to its retroperitoneal location, subtle clinical presentation, and nonspecific early imaging findings. This case report highlights the critical role of contrast-enhanced CT in the diagnostic pathway. The identification of direct signs such as intravenous contrast endoluminal extravasation is an unusual CT finding that may raise suspicion for significant duodenal wall injury, including perforation, particularly in the appropriate clinical and traumatic context. However, this finding is not specific for perforation and may also reflect active intraluminal hemorrhage or other vascular or traumatic mucosal abnormalities. Recognition of this sign, in conjunction with other CT findings and systematic assessment of the entire duodenum, may facilitate early diagnosis and appropriate surgical management.
